# Near‐Infrared Spectroscopy Prediction of Dry Matter and Starch Content in Cassava Using Optimized Calibration Models

**DOI:** 10.1111/1750-3841.70704

**Published:** 2025-11-22

**Authors:** Paulo Henrique Ramos Guimarães, Massaine Bandeira e Sousa, Marcos de Souza Campos, Cinara Fernanda Garcia Morales, Eder Jorge de Oliveira

**Affiliations:** ^1^ Embrapa Mandioca e Fruticultura Cruz das Almas Bahia Brazil

**Keywords:** crop modeling, high‐throughput phenotyping, root quality traits, selection, spectral data analysis

## Abstract

Dry matter content (DMC) and starch content (StC) are key quality traits in cassava breeding, yet traditional phenotyping methods are time‐consuming and limit scalability. This study aimed to develop and compare predictive models for DMC and StC using near‐infrared (NIR) spectroscopy, evaluating two devices—a benchtop spectrometer (Büchi NIRFlex N‐500; 1000–2500 nm) and a portable device (QualitySpec Trek; 350–2500 nm)—and assessing the influence of sample type (fresh vs. processed). A total of 3,391 cassava clones from the Embrapa breeding program were analyzed from 2018 to 2023. Reference values were obtained via gravimetric analysis (DMCg), oven drying (DMCo), and manual StC extraction. Spectral data were used to train and validate models using Partial Least Squares (PLS), k‐Nearest Neighbors (KNN), and eXtreme Gradient Boosting (XGB). PLS consistently delivered the highest predictive accuracy across traits and devices. KNN slightly outperformed PLS for DMCg using the benchtop device, while XGB was comparable to PLS in select scenarios (e.g., StC with the benchtop: 0.88 vs. 0.89; DMCo with the portable: 0.92 vs. 0.95). Processed samples yielded higher model accuracy than fresh ones. The portable NIR device showed better performance with processed samples and even surpassed the benchtop for DMCg and StC in external validation (0.74 and 0.76 vs. 0.71 and 0.72, respectively). Overall, processed sample preparation significantly improved model performance, and the portable spectrometer proved to be a practical, accurate, and scalable alternative for high‐throughput phenotyping in cassava breeding.

## 1 Introduction

Accurate quantification of root quality traits is essential for effective genotype selection in cassava (*Manihot esculenta*) breeding programs. Among these traits, starch content (StC) and dry matter content (DMC) are particularly important, as they determine root processing efficiency and commercial value (Bantadjan et al. 2020). Starch, comprising up to 80% of the dry matter, is composed mainly of amylopectin (∼83%) and amylose (∼17%) (Gomes et al. 2005; Promthong et al. 2005), with minor contributions from proteins (Ngiki et al. 2014). Its wide industrial application—from food and bioenergy to biodegradable plastics and cosmetics—has intensified global demand (Olivato 2024; Wang et al. 2022). However, expanding cassava cultivation to meet this demand is constrained by limited arable land, competition with other crops, and growing environmental concerns (Vilpoux and Silveira Junior 2023). A sustainable alternative is to improve root quality traits through breeding, enhancing yield per unit area without requiring land expansion (Mbanjo et al. 2022). Yet, a major challenge lies in phenotyping DMC and StC accurately and efficiently at scale.

Traditional methods such as gravimetry and oven drying are widely used in breeding programs and industry (Kawano et al. 1987; Vasconcelos et al. 2017). While cost‐effective, they are labor‐intensive, low‐throughput, and prone to variability due to genotype, plant age, and environmental conditions (Pola et al. 2020; Silva et al. 2023). Transporting root samples to centralized facilities for processing further complicates logistics and introduces risks of sample degradation (Hershberger et al. 2022).

In this context, high‐throughput phenotyping (HTP) technologies have gained attention for enabling rapid and large‐scale evaluations (Cobb et al. 2013; Reynolds et al. 2020). Among them, near‐infrared spectroscopy (NIR) has emerged as a powerful, non‐destructive method for predicting biochemical traits. NIR captures the interaction of light with chemical bonds (C–H, O–H) to infer compound composition, offering rapid predictions for multiple traits simultaneously (Cortés et al. 2019; Osborne 2006).

NIR has been successfully applied to assess cassava traits such as cooking time, carotenoid levels, and cyanogenic potential (Chaiareekitwat et al. 2024; Sánchez et al. 2014). For DMC and StC, prediction accuracy varies widely across devices and statistical models. For example, Sánchez et al. (2014) achieved high DMC prediction accuracy ($R^{2}$ = 0.96) with a benchtop spectrometer and PLS regression, while lower performance was observed using handheld or low‐cost devices (Hershberger et al. 2022; Tipsod et al. 2024).

Portable and miniaturized NIR devices, such as the QualitySpec Trek (QST) and SCiO, offer practical advantages for field‐based phenotyping and have shown promising results (Ikeogu et al. 2017; Mbanjo et al. 2022). Still, prediction performance depends not only on device resolution and range but also on the type and preparation of the sample. Mashed samples provide more consistent spectral signals but are labor‐intensive, whereas fresh root analysis is more suitable for field deployment (Beć et al. 2022; Ikeogu et al. 2017).

While predictive models for DMC and StC have been extensively tested in African cassava populations, their validation in Brazilian germplasm remains limited. Given the genetic diversity and distinct environmental conditions, tailored calibration is essential.

This study aimed to (i) develop and validate NIR‐based predictive models for dry matter and starch content in Brazilian cassava clones; (ii) compare the performance of two spectrometers—a benchtop (NIRFlex N‐500) and a portable (QualitySpec Trek); and (iii) evaluate the impact of sample type (fresh vs. mashed) on model accuracy.

## 2 Material and Methods

### 2.1 Plant Material

**FIGURE 1:**
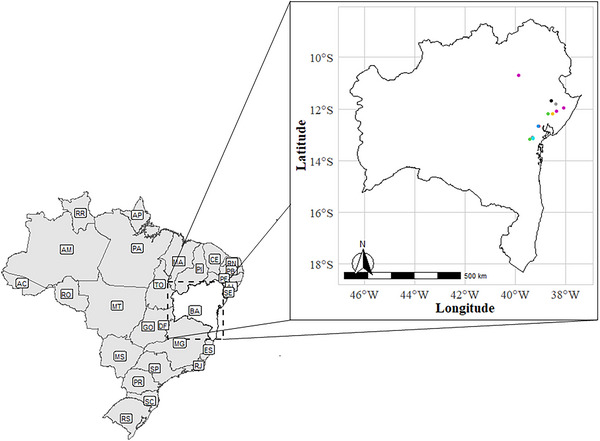
Geographical distribution of specific locations of the field trials conducted in Bahia State, Brazil.

A panel of 3,840 clones from the Embrapa Mandioca e Fruticultura breeding program in Cruz das Almas, Bahia, Brazil (12°39′25″ S, 39°07′27″ W, 226 m altitude), was field‐phenotyped across 50 trials conducted from 2018 to 2023. These clones represented diverse populations from multiple breeding stages, including clonal evaluation trials (CET), preliminary yield trials (PYT), advanced yield trials (AYT), uniform yield trials (UYT), and unimproved germplasm (BAG). Trials were conducted at 15 experimental sites across Bahia State (Figure 1). The region has a hot and humid tropical climate (Aw/Am, Köppen classification), with average annual rainfall of 1,170 mm (range: 900 mm–1,300 mm) and mean annual temperature around 25°C.

### 2.2 Experimental Conditions

Trials were conducted under rainfed conditions during the rainy season (May–July). A randomized complete block design (RCBD) with two or three replicates was used for CET, PYT, and AYT, while an augmented block design was used for BAG trials, including 10 standard checks per block. Stem cuttings (15–20 cm, 5–7 buds) were planted at 0.90 m × 0.80 m spacing. Fertilization included 40 kg·ha^−1^ of P_2_O_5_ at planting and a topdressing of 30 kg·ha^−1^ of N and 45 kg·ha^−1^ of K_2_O at 50–60 days, following Souza et al. (2006).

Plots were harvested 11–12 months after planting. At harvest, 6–10 healthy roots per clone were randomly selected, cleaned, trimmed, and assessed for trait analysis and spectral data. Roots were selected based on size, shape, and absence of pests or disease. Three traits were evaluated: (i) DMC (gravimetric, DMCg, %, *n* = 2,432 clones): ∼5 kg of cleaned roots were weighed in air and water to determine specific gravity, following Kawano et al. (1987); (ii) DMC (oven‐dry, DMCo, %, *n* = 3,299 clones): ∼200 g of grated tissue from various root sections were dried at 90°C for ∼72 h until constant weight (Carvalho et al. 2022); (iii) Starch content (StC, %, *n* = 1,132 clones): determined using protocols from Sánchez et al. (2009) and Vasconcelos et al. (2017).

### 2.3 Spectral Data Collection and Analysis

Spectral data were collected in a laboratory at a controlled room temperature of 22°C  ±  1°C using ultraviolet‐visible and near‐infrared (NIR) spectrophotometry. Two devices were employed: a benchtop Büchi NIRFlex N‐500 spectrometer (Büchi, Flawil, Switzerland), hereafter referred to as NIR.N500, and the portable QualitySpec Trek model S‐10016, hereafter referred to as NIR.QST. The NIR.N500 operates in diffuse reflectance mode, covering a spectral range of 1000–2500 nm (equivalent to 10,000–4,000 cm^−1^), with a spectral resolution of 8 cm^−1^ interpolated to 4 cm^−1^, resulting in 1,501 data points per spectrum. It employs a polarization interferometer with TeO_2_ wedges, a tungsten‐halogen lamp as the light source, and an InGaAs detector, ensuring high‐precision data acquisition. In contrast, the NIR.QST device measures spectra in diffuse reflectance across a broader range (350–2500 nm), with a spectral resolution of 9.8 nm at 1400 nm. It utilizes three detectors: a 512‐element silicon array (350–1000 nm), an InGaAs photodiode (1001–1785 nm), and a second InGaAs photodiode (1786–2500 nm). This device is equipped with an internal quartz tungsten halogen bulb (color temperature 2870 ± 33 K) and an integrated grayscale reference for automatic optimization and wavelength calibration. The measurement window, approximately 1 cm in diameter, is internally illuminated and designed to minimize specular reflections, enhancing measurement accuracy.

For spectral data collection, fifteen roots were randomly selected from each experimental plot. These roots were harvested from three competitive plants (five healthy roots per plant) and had diameters ranging from 4 to 7 cm. After sorting, the roots were washed under running water and peeled. From each root, two sections were taken from the central region and one each from the proximal and distal regions, all approximately 10 cm in length, with diameters varying according to genotype. For the fresh root samples, spectra were collected directly from each of these sections (central, proximal, and distal) to capture longitudinal variability along the root axis. The second central section from each root was cut into pieces approximately 3 × 10 mm in size using kitchen knives. These pieces were then cooked and homogenized in a food processor. Approximately 8 grams of the resulting mash were transferred to quartz cups suitable for spectral analysis, which were then placed against the reading window of the spectrometer. Spectral measurements were performed using two NIR devices. The portable NIR.QST unit was used for both fresh and mashed samples, while the NIR.N500 device was used exclusively for the mashed samples. To ensure robust data representation, six replicates per plot were analyzed, encompassing all sample types and measurement methods.

The preprocessed spectral data were utilized to evaluate heritability of each wavelength across all trials. Variance components were estimated using the following linear mixed model: $y=X_{b}+Z_{g}+e$, where y: is the wavelength reflectance data, b: is the fixed effects associated with blocks/repetitions, and g and e are the random effects due to genotype and error, respectively. X and Z correspond to the incidence matrices for the fixed and random effects respectively within the mixed model. It was assumed that all random effects followed a normal distribution, expressed as g∼ N(0, $\sigma_{g}^{2}$) and N(0, $\sigma_{e}^{2}$). The broad‐sense heritability ($H^{2}$) was estimated as following: $H^{2}=\frac{\sigma_{g}^{2}}{\sigma_{g}^{2}+\frac{\sigma_{e}^{2}\;}{nRep}}$, where, $\sigma_{g}^{2}$ is the genotypic variance, $\sigma_{e}^{2}$ is the error variance and nRep:is the mean number of repetitions for one genotype in the trial. The analysis was performed using the *lme4* package (Bates et al. 2015) in R software (R Core Team 2024). The heritability estimates of the entire measured NIR spectrum were plotted using the *ggplot()* function from the *ggplot2* package (Wickham 2009).

### 2.4 Spectral Data Pre‐Treatment and Calibration Models

To reduce the effects of light scattering, the raw spectral data were initially pre‐processed using a Savitzky‐Golay (SG) filter with a window size of 11, a third‐order polynomial, and the application of the first derivative. This method smooths spectral data by fitting successive subsets of data points to a polynomial, preserving essential spectral features such as peaks and troughs while reducing noise (Savitzky and Golay 1964). To further mitigate baseline drift and random noise, the SG filter was combined with the gap‐segment derivative (window size = 11, segment size = 7, first derivative), which computes derivatives between data points separated by a fixed gap, enhancing small spectral variations. Additionally, we applied the Standard Normal Variate (SNV) transformation to correct for multiplicative scatter effects by normalizing each spectrum to zero mean and unit variance. These three preprocessing steps—SG smoothing, gap‐segment derivative, and SNV—were implemented using the savitzkyGolay(), gapDer(), and standardNormalVariate() functions, respectively, from the prospectr package (Stevens and Ramirez‐Lopez 2013).

Only trials showing spectral heritability values above 0.3 and common to both NIR.QST and NIR.N500 devices were retained for subsequent model development. To reduce bias introduced by extreme values, outliers were excluded based on a spectral standard deviation (SD) threshold. Specifically, SDs were calculated per observation across wavelengths, and observations with SD values exceeding 1.5 times the overall trial SD were considered outliers and removed. The cleaned spectral data were structured into a predictor matrix (X) comprising the reflectance values, while the response vector (Y) included the measured quality traits: DMCg, DMCo, and StC. The dataset was then randomly split into a training set (80%) and an external validation set (20%) for model evaluation.

Three regression models were selected for their relevance and complementary strengths in NIR calibration for agricultural applications. The first model was Partial Least Squares (PLS) regression, also known as projection to latent structures, which simultaneously models the predictor (X) and response (Y) matrices to identify latent variables in X that best explain the variance in Y. The resulting components, referred to as PLS factors, are analogous to principal components but optimized for prediction (Abdi 2010). The second model was the k‐Nearest Neighbors (KNN) algorithm, a simple, non‐parametric technique widely used in machine learning. While KNN is most commonly applied to classification, it also has a regression variant (KNN regression) that predicts continuous outcomes based on the values of the nearest neighbors. KNN operates on the premise that similar samples are located near each other within the feature space. To classify a new sample, the algorithm calculates the distance—commonly Euclidean—between the sample and all training instances, selecting the k closest neighbors. The sample is then assigned to the class most frequently represented among these neighbors through a majority vote. The optimal value of k is determined by assessing predictive accuracy across various k values (Mucherino et al. 2009).

The third model tested was eXtreme Gradient Boosting (XGB), a high‐performance implementation of the gradient boosting decision tree (GBDT) algorithm (Chen and Guestrin 2016). Unlike traditional random forest models (Svetnik et al. 2003), XGB incorporates a regularization term that reduces overfitting, enhances prediction accuracy, and accelerates model training (Luckner et al. 2017). To fine‐tune the model, we selected a set of hyperparameters based on previous performance evaluations. The number of boosting iterations (nrounds) was set to 200, and the maximum tree depth (max_depth) was limited to 5 to control model complexity. The learning rate (eta) was fixed at 0.3 to balance training speed and convergence. A regularization parameter (gamma) of 5 was used to ensure a minimum loss reduction was required before further splitting a tree node. To reduce overfitting and improve generalization, 70% of the training samples (subsample = 0.7) and 70% of the features (colsample_bytree = 0.7) were randomly selected for each tree. The minimum sum of instance weight needed in a child node (min_child_weight) was set to 1, providing flexibility in tree growth. This setup aimed to optimize predictive accuracy while maintaining model stability. All model analyses were conducted using the *caret* package in R (Kuhn 2008).

Model performance was evaluated on the training set using a repeated cross‐validation strategy, consisting of five repetitions of 5‐fold cross‐validation. Outliers within the training data were identified based on residuals from an initial model run. To enhance model calibration, a threshold was applied to exclude the 20% of data points with the highest residuals, thereby reducing the influence of extreme values on model performance. Following this refinement, models were retrained on the filtered dataset to improve robustness and predictive accuracy. The goodness of fit of the training models, as well as external validation performance, were assessed using several statistical metrics: Pearson's correlation coefficient (R) between observed and predicted values (also referred to as prediction accuracy), the coefficient of determination extracted from each model ($R^{2}$), bias, and the root mean squared error (RMSE).

## 3 Results

### 3.1 NIR Spectral Heritability

**FIGURE 2:**
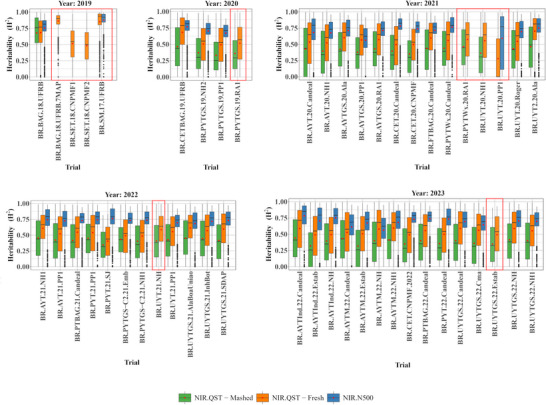
Broad‐sense heritability ($H^{2}$) of cassava root near‐infrared (NIR) spectra across multiple field trials conducted over different years and locations. The spectra were measured using two different NIR devices (NIR.QST and NIR.N500) and analyzed for two sample preparation types (mashed and fresh cassava roots). The boxplots highlighted in red represent trials that are not available across all devices (NIR.QST and NIR.N500) and sample types (mashed and fresh).

Broad‐sense heritability ($H^{2}$) estimates for NIR spectra ranged from 0.28 to 0.88, reflecting variation across trials (Figure 2), devices (NIR.QST and NIR.N500), and sample types (mashed and fresh). Considerable genotypic variation was captured among cassava clones. The benchtop NIR.N500 consistently showed higher heritability (0.56–0.88) than the portable NIR.QST (0.32–0.68), especially for mashed samples. Interestingly, for fresh samples, NIR.QST values (0.28–0.86) were more comparable to NIR.N500. Across years and trials, the trend was: $H^{2}$ (NIR.N500) > $H^{2}$ (NIR.QST–fresh) > $H^{2}$ (NIR.QST–mashed). Except for the BR.UYT.20.PP1 trial (Figure 2C), NIR.N500 values were stable across wavelengths, while NIR.QST trials showed more variability, regardless of sample type. The BR.UYT.20.PP1 trial (NIR.QST, fresh) showed the lowest $H^{2}$ (0.28).

Although several trials—including BR.BAG.18.UFRB.7MAP, BR.SET.18.CNPMF1, BR.SET.18.CNPMF2, BR.SM.17.UFRB (Figure 2A), BR.PYTGS.19.RA1 (Figure 2B), BR.PYTWx.20.RA1, BR.UYT.20.PP1, BR.UYT.20.NH1 (Figure 2C), BR.UYT.21.NH (Figure 2D), and BR.UYTGS.22.Estab (Figure 2E)—showed $H^{2}$ above 0.4, they were excluded due to incomplete evaluation with both devices and sample types, limiting prediction comparisons.

### 3.2 Assessment of Prediction Accuracy Across Instruments and Models

**FIGURE 3:**
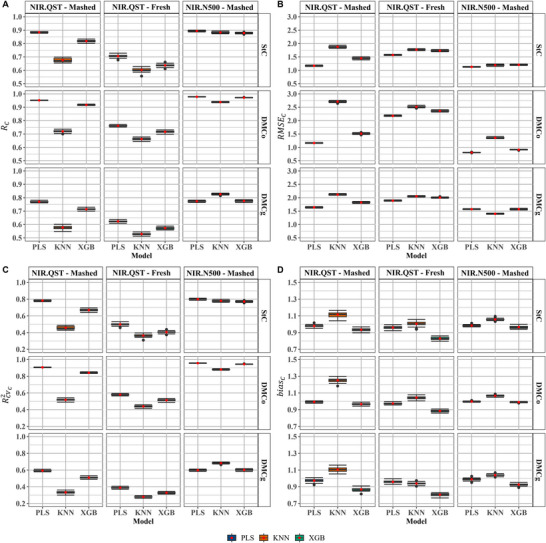
Calibration performance based on near‐infrared (NIR) spectra collected using the Büchi NIRFlex N‐500 (NIR.N500) and the QualitySpec Trek (NIR.QST) instruments. Performance metrics are shown for different calibration models and root sample types (mashed and fresh). (A) Prediction accuracy of cross‐validation ($R_{C}$); (B) Root mean square error of cross‐validation ($RMSE_{C}$); (C) Coefficient of determination of cross‐validation ($R_{CV}^{2}$); (D) Calibration bias ($bias_{C}$). StC—starch content; DMCo—dry matter content determined by the oven‐drying method; DMCg—dry matter content determined by the gravimetric method.

The predictive models—PLS, KNN, and XGB—were assessed using the following statistical metrics: prediction accuracy ($R_{C}$, Figure 3A), root mean squared error ($RMSE_{C}$, Figure 3B), coefficient of determination of cross‐validation ($R_{CV}^{2}$, Figure 3C), and prediction bias ($bias_{C}$, Figure 3D). The influence of sample type on prediction performance was particularly pronounced in models calibrated with spectra obtained from the NIR.QST device (Figure 3A). In general, models developed using mashed samples outperformed those based on fresh samples, exhibiting higher $R_{C}$ and $R_{CV}^{2}$ values, indicating superior predictive accuracy and model robustness.

#### 3.2.1 Starch Content (StC)

Prediction accuracy ($R_{C}$) for StC varied significantly across calibration models, sample types, and NIR devices (Figure 3A). The NIR.N500 outperformed the NIR.QST in all models, yielding higher $R_{C}$ and $R_{CV}^{2}$ values and lower $RMSE_{C}$ values (Figure 3). Among models using NIR.N500 data, the PLS model showed the best performance, with $R_{C}$ = 0.89, $R_{CV}^{2}$ = 0.80, $RMSE_{C}$= 1.13, and $bias_{C}$ = 0.98 (Figure 3). Although the KNN and XGB models also showed relatively high $R_{C}$ values (0.88 each) and comparable $R_{CV}^{2}$ values (0.78 and 0.77, respectively), their $RMSE_{C}$ values were slightly higher (1.19 and 1.21), and $bias_{C}$ was moderately greater for KNN (1.06) compared to XGB (0.97) (Figure 3). Similarly, for mashed samples analyzed with the NIR.QST, the PLS model again showed the best prediction performance, with $R_{C}$ = 0.88, $RMSE_{C}$= 1.17, $R_{CV}^{2}$ = 0.78, and $bias_{C}$ = 0.98 (Figure 3). The XGB model also performed reasonably well ($R_{C}$ = 0.82), but its prediction metrics were inferior to those of PLS ($RMSE_{C}$= 1.45, $R_{CV}^{2}$ = 0.67, $bias_{C}$ = 0.93) (Figures 3B–D). The KNN model had the poorest performance among the three, with $R_{C}$ = 0.68, $RMSE_{C}$= 1.88, $R_{CV}^{2}$ = 0.46, and $bias_{C}$ = 1.12. When using fresh samples, all models exhibited reduced predictive performance compared to mashed samples. The KNN and XGB models had the lowest prediction accuracy, with $R_{C}$ = 0.60, $RMSE_{C}$= 1.77, $R_{CV}^{2}$ = 0.36, and $bias_{C}$ = 1.01 for KNN; and $R_{C}$ = 0.64, $RMSE_{C}$ = 1.73, $R_{CV}^{2}$ = 0.41, and $bias_{C}$ = 0.83 for XGB (Figure 3).

Among the models using fresh samples, PLS again showed superior predictive performance, achieving the highest $R_{C}$ (0.71) and the lowest $RMSE_{C}$ (1.57), along with $R_{CV}^{2}$ = 0.50 and $bias_{C}$ = 0.96, thus confirming its robustness across different sample types (Figures 3A‐3D).

#### 3.2.2 DMC by Oven‐Drying Method (DMCo)

Overall, the predictions of DMCo showed that the NIR.N500 device had a higher predictive performance compared to the NIR.QST. Prediction accuracy ($R_{C}$) ranged from 0.94 to 0.98, while the root mean square error of calibration ($RMSE_{C}$) varied between 0.81 and 1.36 (Figures 3A and 3B). Among the evaluated models, PLS and XGB demonstrated the highest accuracies, with $R_{C}$ values of 0.98 and 0.97, respectively, and $R_{CV}^{2}$ of 0.96 and 0.94, respectively (Figures 3A and 3C). The PLS model showed a slight advantage over XGB, presenting a lower $RMSE_{C}$ (0.81 vs. 0.91) (Figure 3B). Both models exhibited a positive bias ($bias_{C}$ = 1.00 and 0.99), indicating a mild tendency to overestimate predicted values (Figure 3D). While the KNN model also achieved a high $R_{C}$ of 0.94 and $R_{CV}^{2}$ of 0.88 (Figures 3A and 3C), it presented a higher $RMSE_{C}$ (1.36) (Figure 3B), suggesting comparatively lower predictive performance.

Sample processing significantly influenced model performance when using the NIR.QST device. For fresh samples, the models yielded lower $R_{C}$ values and higher $RMSE_{C}$ values, indicating reduced predictive accuracy. For instance, the PLS model yielded $R_{C}$ = 0.76, $R_{CV}^{2}\;$= 0.58, and $RMSE_{C}$= 2.18, reflecting poor predictive capacity (Figures 3A and 3B). The KNN and XGB models also exhibited limited performance with fresh samples, producing $R_{C}$ values between 0.66 and 0.72 and $RMSE_{C}$ values of 2.52 and 2.36, respectively (Figures 3A and 3B). In contrast, mashed samples improved model performance. The PLS and XGB models achieved $R_{C}$ values ranging from 0.92 to 0.95 and $R_{CV}^{2}$ values between 0.84 and 0.91 (Figures 3A and 3C). The PLS model again presented a lower $RMSE_{C}$ (1.16) compared to XGB (1.52) (Figure 3B). Although the KNN model achieved an $R_{C}$ of 0.72, its $RMSE_{C}$ was the highest among the models (2.71), and it showed the largest prediction $bias_{C}$ (1.25), while PLS and XGB maintained lower bias values (ranging from 0.97 to 0.99) (Figure 3D).

#### 3.2.3 DMC by Gravimetric Method (DMCg)

Among all traits assessed, DMCg exhibited the lowest predictive performance. $R_{C}$ values ranged from 0.53 to 0.83 (Figure 3A), indicating lower model and device performance compared to DMCo. The NIR.QST device showed especially poor performance with fresh samples, with $R_{C}$ values between 0.53 and 0.62 and $R_{CV}^{2}$ ranging from 0.28 to 0.39 (Figures 3A and 3C). $RMSE_{C}$ values were also relatively high, ranging from 1.89 to 2.05 (Figure 3B). Among the models, PLS ($R_{C}$ = 0.62) and XGB ($R_{C}$ = 0.57) outperformed KNN ($R_{C}$ = 0.53) (Figure 3A), although cross‐validation performance remained low across all models ($R_{CV}^{2}$ of 0.28 for KNN, 0.33 for XGB, and 0.39 for PLS) (Figure 3C). $bias_{C}$ values for fresh samples varied from 0.81 (XGB) to 0.96 (PLS) (Figure 3D).

Using mashed samples improved model predictions for DMCg with the NIR.QST device. PLS and XGB yielded higher $R_{C}$ values (0.77 and 0.71, respectively), while $R_{CV}^{2}$ values ranged from 0.51 (XGB) to 0.59 (PLS) (Figure 3C). $RMSE_{C}$ values, however, remained high (1.64 for PLS and 1.82 for XGB) (Figure 3B). $bias_{C}$ values for mashed samples were similar to those from fresh samples, with XGB and PLS showing values of 0.87 and 0.97, respectively (Figure 3D). In contrast, KNN performed poorly, with $R_{C}$ = 0.58, $RMSE_{C}$= 2.12 and $R_{CV}^{2}\;$= 0.33 (Figures 3A–B). The NIR.N500 device showed the highest predictive performance for DMCg. Notably, the KNN model achieved the best results for this trait, with the highest $R_{C}$ (0.83), $R_{CV}^{2}$ (0.68), and the lowest $RMSE_{C}$ (1.40) (Figures 3A, 3C, and 3B). XGB and PLS also performed well, both with $R_{C}$ values between 0.77 and 0.78, $RMSE_{C}$ of 1.57, and $R_{CV}^{2}$ values around 0.60. Despite KNN's superior $R_{C}$ and $RMSE_{C}$, it exhibited a higher $bias_{C}$ (1.04), indicating a slight overestimation. In contrast, PLS and XGB had lower $bias_{C}$ values (0.99 and 0.93, respectively) (Figure 3D).

### 3.3 External Validation of Prediction Models

#### 3.3.1 Starch Content (StC)

The same statistical parameters used for evaluating the prediction quality of calibration model samples were applied to the external validation set, with modified nomenclature to distinguish them from calibration metrics by adding the suffix “v”. These included prediction accuracy ($R_{v}$), root mean squared error ($RMSE_{v}$), coefficient of determination for prediction ($R_{v}^{2}$), and prediction bias ($bias_{v}$).

In the external validation of the calibration model for StC, the predictive performance varied depending on the NIR device, sample type, and prediction model. The best performance was observed using the NIR.QST device with mashed samples and the PLS model, yielding $R_{v}$ = 0.76, $R_{v}^{2}$ = 0.58, $RMSE_{v}$ = 1.87, and $bias_{v}$ = 0.82. The NIR.N500, under the same conditions, showed slightly lower performance ($R_{v}$ = 0.75, $R_{v}^{2}$= 0.56, $RMSE_{v}$ = 1.93, $bias_{v}$ = 0.80). Although the PLS model demonstrated higher predictive accuracy with both devices for mashed samples, sample type notably influenced prediction quality.

**FIGURE 4:**
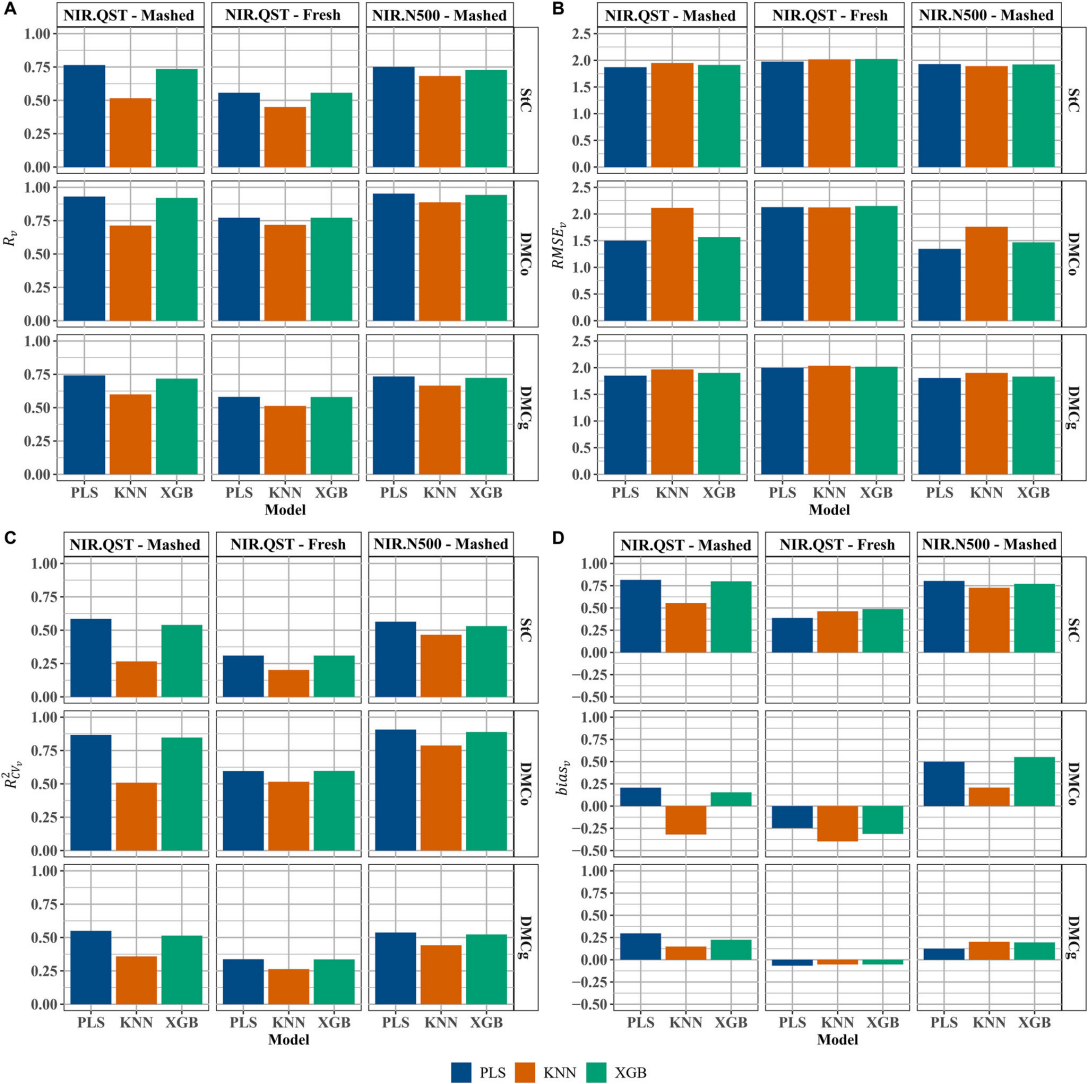
External validation performance of calibration models based on near‐infrared (NIR) spectra collected using two instruments: the Büchi NIRFlex N‐500 (NIR.N500) and the QualitySpec Trek (NIR.QST). Panels show (A) predictive accuracy ($R_{v}$), (B) root mean squared error of prediction ($RMSE_{v}$), (C) coefficient of determination for prediction ($R_{v}^{2}$), and (D) prediction bias ($bias_{v}$), across different sample types (mashed and fresh) and target traits. Evaluated traits include starch content (StC), dry matter content by the oven‐drying method (DMCo), and dry matter content by the gravimetric method (DMCg).

When fresh samples were analyzed, the $R_{v}$ (0.56) and $R_{v}^{2}$ (0.31) values decreased, and $RMSE_{v}$ increased to 1.97 compared to mashed samples for both devices (Figures 4A and 4B). The XGB model showed slightly lower predictive capacity than PLS, with $R_{v}$ = 0.73 for both devices with mashed samples. It also exhibited low variation between devices in terms of $R_{v}^{2}$ (0.54 and 0.53), $RMSE_{v}$ (1.91 and 1.92), and $bias_{v}$ (0.80 and 0.77) (Figures 4B–D). In contrast, the KNN model showed the poorest performance across all devices and sample types, with $R_{v}$ ranging from 0.45 to 0.68 and $R_{v}^{2}$ between 0.20 and 0.47. $RMSE_{v}$ values were higher, especially for the NIR.N500 (1.89) and the NIR.QST using mashed (1.95) and fresh (2.02) samples, confirming the limitations of KNN in this context (Figure 4B).

#### 3.3.2 DMC by Oven‐Drying (DMCo)

For DMCo, the NIR.N500 device generally demonstrated the highest predictive capacity, particularly when combined with the PLS and XGB models. These models achieved the highest $R_{v}$ values (0.95 and 0.94, respectively) and $R_{v}^{2}$ (0.91 and 0.89), along with the lowest prediction errors ($RMSE_{v}$ = 1.35 and 1.47) and relatively low biases ($bias_{v}$ = 0.50 and 0.55) (Figures 4A–D). Similar results were observed with the NIR.QST device and mashed samples, where both PLS and XGB models also showed high $R_{v}$ values (0.93 and 0.92) and $R_{v}^{2}$ (0.87 and 0.85). The PLS model performed slightly better than XGB, reflected by a lower $RMSE_{v}$ (1.50 vs. 1.57) (Figure 4B). Although the KNN model achieved relatively high $R_{v}$ values (0.71 for NIR.QST and 0.89 for NIR.N500), its prediction errors ($RMSE_{v}$ = 2.11 and 1.76, respectively) were higher than those observed for the PLS and XGB models using the same devices and sample types (Figures 4A and 4B). Conversely, the analysis of fresh samples using NIR.QST showed inferior performance compared to mashed samples. For these, the PLS, XGB, and KNN models yielded similar $R_{v}$ values (0.77 for PLS and XGB; 0.72 for KNN) and $R_{v}^{2}$ values (0.60 for PLS and XGB; 0.52 for KNN). However, all models exhibited higher $RMSE_{v}$ values (2.13, 2.12, and 2.15, respectively) and negative $bias_{v}$ values (‐0.25, ‐0.40, and ‐0.31), indicating a systematic underestimation of predictions (Figures 4A–D).

#### 3.3.3 DMC by Gravimetric Method (DMCg)

For DMCg, the PLS and XGB models exhibited similar predictive performance when applied to NIR spectra from both the NIR.N500 and NIR.QST devices using mashed samples (Figure 4). In both cases, the models produced $R_{v}$ values ranging from 0.74 for PLS to 0.73 for XGB, regardless of the device (Figure 4A). $RMSE_{v}$ and $R_{v}^{2}$ values showed little variation between the two models, though PLS slightly outperformed XGB, with lower $RMSE_{v}$ values (1.84 for NIR.QST and 1.79 for NIR.N500) and higher $R_{v}^{2}$ (0.55 and 0.54, respectively) (Figures 4B and 4C). In contrast, XGB yielded $RMSE_{v}$ values of 1.87 and 1.80, and $R_{v}^{2}$ of 0.53 and 0.54 for the NIR.QST and NIR.N500, respectively (Figures 4B and 4C). The KNN model showed inferior performance compared to PLS and XGB. Using NIR.QST with mashed samples, KNN had $R_{v}$ = 0.61, $RMSE_{v}$ = 1.95, $R_{v}^{2}$ = 0.37, and $bias_{v}$ = 0.16. With NIR.N500, performance slightly improved ($R_{v}$ = 0.68, $RMSE_{v}$ = 1.87, $R_{v}^{2}$ = 0.46, and $bias_{v}$ = 0.19) (Figures 4A–D). In external validation with fresh samples analyzed using the NIR.QST, all models showed reduced performance compared to mashed samples (Figure 4). The PLS and XGB models maintained similar performance ($R_{v}$ = 0.58 for PLS and 0.60 for XGB, $R_{v}^{2}$ = 0.34 and 0.36 for XGB) (Figures 4A–C), with slight differences in $RMSE_{v}$ (1.99 for PLS and 1.98 for XGB), suggesting a minor advantage for XGB (Figure 4B). $bias_{v}$ values were also slightly different: ‐0.08 for PLS and ‐0.05 for XGB (Figure 4D). Once again, the KNN model demonstrated the lowest predictive accuracy, with $R_{v}$ = 0.52, $RMSE_{v}$ = 2.02, $R_{v}^{2}$ = 0.27, and $bias_{v}$ = ‐0.06 (Figures 4A–D).

## 4 Discussion

### 4.1 Assessing the Stability of Spectral Heritability in Cassava

Spectral heritability ($H^{2}$) estimates ranged from 0.28 to 0.88, showing substantial variation across trials, instruments (NIR.QST and NIR.N500), and sample types (mashed and fresh) (Figure 2). The NIR.N500 showed greater temporal consistency with high $H^{2}$ values (0.56–0.88), outperforming the NIR.QST—especially in mashed samples, where $H^{2}$ ranged 0.32–0.68. This agrees with Mbanjo et al. (2022), who reported $H^{2}$ between 0.29 and 0.88 using mashed samples from 344 cassava clones scanned by the SCiO device. Our NIR.QST estimates for fresh samples ranged 0.28–0.86. In addition, Sousa et al. (2024), evaluating 888 cassava clones from Embrapa, found slightly lower $H^{2}$ values for NIR.QST mashed samples (0.18–0.74) and marginally lower values for NIR.N500 (0.32–0.84) compared with our study (Figure 2).

Spectral heritability followed: $H^{2}$ (NIR.N500) > $H^{2}$ (NIR.QST—fresh) > $H^{2}$ (NIR.QST—mashed), with higher variability in NIR.QST mashed samples (Figure 2). This likely reflects the superior spectral resolution and refined calibration of the NIR.N500, minimizing noise and improving precision (Kirchler et al. 2017; Pasquini 2018). Benchtop spectrometers operate under controlled conditions, ensuring stable light‐sample interaction, while portable devices are more prone to variability from sensor positioning, sample contact, and environment (Hernández‐Jiménez et al. 2024; Sorak et al. 2012).

Differences between fresh and mashed samples arise from structural changes during processing. Fresh samples retain cellular integrity, allowing uniform light reflection. Mashing disrupts tissue, altering scattering and moisture distribution, which affects near‐infrared absorption bands linked to water content (Cozzolino and Murray 2004; Gorji et al. 2024). Instruments with better control of reflection/scattering, like benchtop spectrometers, yield more reliable heritability estimates. Stable spectral signatures are critical for phenotyping and breeding; selecting wavelengths with high heritability improves model robustness and predictive stability across environments (Posada et al. 2009).

### 4.2 Optimizing NIR Predictions: The Role of Sample Preparation and Spectrometer Type

Sample type directly affected calibration model performance with NIR.QST, significantly impacting $R_{C}$ and $\mathrm{RMS}E_{C}$ (Figure 3A). Mashed samples consistently showed superior prediction ($R_{C}$: 0.58–0.95; $\mathrm{RMS}E_{C}$: 1.16–2.71) compared to fresh ($R_{C}$: 0.53–0.76; $\mathrm{RMS}E_{C}$: 1.57–2.52). This gap was greater with NIR.N500, which achieved the best results overall ($R_{C}$: 0.77–0.98; $\mathrm{RMS}E_{C}$: 0.81–1.57) (Figures 3A and 3B). $R_{CV}^{2}$ varied with sample and device: NIR.QST mashed samples had higher $R_{CV}^{2}$ (0.33–0.91) than fresh (0.28–0.58), while NIR.N500 ranged 0.60–0.96 (Figure 3C). This superior performance reflects increased homogeneity in mashed samples, enhancing spectrometer sensitivity and calibration reproducibility (Lafargue et al. 2003; Pasikatan et al. 2001). Ikeogu et al. (2017) similarly found mashed samples improved NIR.QST calibration for DMCo, with $R_{CV}^{2}$ between 0.96 and 0.99 versus 0.86–0.96 for fresh; $R_{CV}^{2}$ was also higher (0.84–0.95 vs. 0.55–0.64). These differences relate to heterogeneous starch and dry matter distribution within roots, with gradients radially and longitudinally affecting concentration (Boerboom 1978; Carvalho et al. 2009; Carvalho et al. 2018).

Despite variations in spectral heritability estimates across devices and sample types (Figure 2), predictive model performance was largely consistent. Models calibrated with NIR.N500 data and those using NIR.QST with mashed samples consistently outperformed those based on fresh NIR.QST samples (Figure 3). Spectral heritability was primarily used as a filtering criterion to select wavelengths potentially linked to phenotypic variation. This targeted approach reduces spectral variables and computational time, simplifying model development (Li et al. 2020a; Wang et al. 2022). The NIR.N500 consistently exhibited superior performance across models. For DMCo prediction, it achieved $R_{C}$ values up to 0.97 (XGB) and 0.98 (PLS) (Figure 3A), with $R_{C}$ ranging from 0.77 to 0.98, $\mathrm{RMS}E_{C}$ between 0.81 and 1.57, and $R_{CV}^{2}$ from 0.60 to 0.96 (Figures 3B and 3C). The NIR.QST, though portable, also yielded competitive results with mashed samples—$R_{C}$ values of 0.92 (XGB) and 0.95 (PLS). On average, mashed samples with NIR.QST showed slightly lower performance ($R_{C}$= 0.58–0.95, $\mathrm{RMS}E_{C}$ = 1.16–2.71, $R_{CV}^{2}$ = 0.33–0.91), confirming that the device, when paired with homogenized samples, can produce robust, accurate predictions.

In contrast, fresh samples analyzed with the NIR.QST showed significantly lower predictive power ($R_{C}$ = 0.53–0.76, $\mathrm{RMS}E_{C}$ = 1.57–2.52, $R_{CV}^{2}$ = 0.28–0.58), highlighting the importance of sample preparation for portable devices. Similar trends were reported by Sousa et al. (2024) for cooking time predictions in 888 cassava clones. The superior performance of benchtop spectrometers like the NIR.N500 is largely due to higher spectral stability, which improves the detection of subtle phenotypic signals (Beć et al. 2022). However, benchtop spectrometers face logistical challenges in breeding programs. Sample handling, labeling, and transport to labs increase labor and costs compared to field‐based methods. The pipeline also demands infrastructure for data storage and processing (Hershberger et al. 2022; Khongkaew et al. 2022). In remote locations, extended transport may compromise sample integrity through moisture loss, discoloration, or fungal decay (Blagbrough et al. 2010; Mahmod and Beeching 2018; Sánchez et al. 2013; Zainuddin et al. 2018).

In this context, portable spectrometers offer major advantages. Their mobility allows rapid phenotyping with minimal delay between collection and analysis, supporting trials in diverse environments. Their lower cost and ease of use make them attractive for resource‐limited breeding programs. Thus, while benchtop systems offer technical advantages, portable devices—especially with effective sample preprocessing—are strategic tools for accelerating superior genotype selection.

### 4.3 Machine Learning Model Comparison for NIR Calibration

NIR spectroscopy has evolved into a fast, nondestructive, low‐cost method for analyzing large datasets, expanding its applications across science and industry (Li et al. 2020b; Pasquini 2003). This study evaluated the predictive performance of PLS, KNN, and XGB models across NIR devices, sample types (fresh and mashed), and target traits (Figure 3).

Overall, PLS showed the highest accuracy, outperforming XGB and KNN under the tested conditions. It also required less computational time and memory, making it ideal for large‐scale applications or limited‐resource environments. An exception was the DMCg prediction in mashed samples with NIR.N500, where KNN outperformed PLS with higher $R_{C}$ and lower $\mathrm{RMS}E_{C}$ (Figures 3A and 3B), suggesting that instance‐based methods can outperform linear models in specific settings (Altman 1992).

PLS's effectiveness lies in its ability to handle multicollinearity—common in spectral data—making it a standard in chemometrics (Rinnan et al. 2009; Wold et al. 2001). XGB also performed well across both NIR devices, supporting its use for modeling nonlinear relationships in spectral data (Chen and Guestrin 2016). PLS was consistently superior, with lower $\mathrm{RMS}E_{C}$, higher $R_{CV}^{2}$, and lower $bias_{C}$ (Figures 3B–3D). Its $\mathrm{RMS}E_{C}$ ranged from 0.81 to 2.18 and was narrower (0.81–1.57) for NIR.N500 spectra—lower than values reported by Mbanjo et al. (2022) using SCiO, NIR.QST, and FOSS XDS ($\mathrm{RMS}E_{C}$ = 1.89). XGB generally performed between PLS and KNN, with $\mathrm{RMS}E_{C}$ values of 0.91–2.36. In some cases (DMCo, DMCg with NIR.N500), its $R_{CV}^{2}$ was comparable to PLS (Figure 3C). Compared to results by Nantongo et al. (2024) in sweet potato roots ($\mathrm{RMS}E_{C}$ = 0.90–0.92 with XGB and RF), XGB in this study performed better.

KNN generally showed lower accuracy and generalization capacity than PLS and XGB, reflected in lower $R_{C}$ and $R_{CV}^{2}$, and higher $\mathrm{RMS}E_{C}$ and $\mathrm{bia}s_{C}$ (Figures 3A–D). Despite occasionally approaching other models—especially with NIR.N500—it showed higher variability and prediction bias. Still, KNN had competitive results predicting DMCo with NIR.N500 ($R_{CV}^{2}$ = 0.88, $\mathrm{RMS}E_{C}$ = 1.36), outperforming values reported by Tavares et al. (2024) for nitrogen in bean leaves ($R_{CV}^{2}$ = 0.80, $\mathrm{RMS}E_{C}$ = 2.89).

An ideal predictive model should yield a $\mathrm{bia}s_{C}$ close to zero, indicating minimal deviation between predicted and reference values (Chaukhande et al. 2024). In this regard, the PLS model displayed the most consistent behavior, with $\mathrm{bia}s_{C}$ values close to zero across all evaluated conditions, suggesting stable and accurate predictions for starch content (StC), DMCo, and DMCg in cassava (Figure 3D). Conversely, KNN showed higher $\mathrm{bia}s_{C}$ values (ranging from 0.94 to 1.25), indicating lower model precision. XGB demonstrated relatively low and stable $\mathrm{bia}s_{C}$ values (0.81 to 0.99), which may offer an advantage in applications requiring low‐bias estimations. Taken together, these findings indicate that while PLS remains the most robust and stable model for NIR calibration, XGB represents a promising alternative in scenarios requiring nonlinear modeling flexibility. Although KNN underperformed in most cases, its occasional strong performance suggests it may still be useful under specific conditions or with further optimization.

### 4.4 External Validation and the Impact of Sample Maceration

Calibration results indicated that mashed samples consistently outperformed fresh ones across devices and models. This was confirmed through external validation (Figure 4). Mashed samples showed $R_{v}$ values from 0.52 to 0.95 and $\mathrm{RMS}E_{v}$ from 1.35 to 2.11, while fresh samples had lower $R_{v}$ (0.45–0.77) and higher $\mathrm{RMS}E_{v}$ (1.97–2.15) (Figures 4A and 4B). Superior performance of mashed samples is attributed to their homogeneity, improving light interaction and spectral consistency. Fresh roots exhibit internal heterogeneity due to biochemical gradients—starch accumulates in the central parenchyma and dry matter near the periderm. Longitudinally, the root base has higher concentrations than the tip (Boerboom 1978; Carvalho et al. 2009; Carvalho et al. 2018). Maceration minimizes these variations, enabling stronger predictive models.

This inherent heterogeneity in fresh roots poses particular challenges for prediction accuracy, especially when using portable devices such as the NIR.QST. This device showed limited predictive power compared to the benchtop NIR.N500 and was especially underwhelming for predicting StC with the KNN model, where it achieved an $R_{v}$ of just 0.45 and an $\mathrm{RMS}E_{v}$ of 2.02 (Figures 4A and 4B). These results reinforce the value of macerated samples in calibration workflows for NIR spectroscopy, particularly when precision is essential.

Among all tested algorithms, the PLS regression model demonstrated the most consistent and reliable predictive performance for external validation samples, regardless of device or sample type. It was especially effective in predicting DMCo using spectra acquired from the NIR.N500 ($R_{v}$ = 0.95; $\mathrm{RMS}E_{v}$ = 1.35; $R_{v}^{2}$ = 0.91), underscoring its robustness. While the XGB model achieved similar results for DMCo using the same device ($R_{v}$ = 0.94; $\mathrm{RMS}E_{v}$ = 1.47; $R_{v}^{2}$= 0.89), PLS retained a slight edge in overall precision (Figures 4A–C). Notably, when paired with the portable NIR.QST and mashed samples, the PLS model also performed well in predicting StC, DMCo, and DMCg, yielding $R_{v}$ values of 0.76, 0.93, and 0.74; $\mathrm{RMS}E_{v}$ values of 1.87, 1.50, and 1.84; and $R_{v}^{2}$ values of 0.58, 0.87, and 0.55, respectively. The corresponding $\mathrm{bia}s_{v}$ were 0.82, 0.21, and 0.29 (Figures 4A–D). Although the NIR.N500 outperformed the NIR.QST in predictive accuracy—achieving slightly higher $R_{v}$ values for DMCo (0.95 vs. 0.93) and lower $\mathrm{RMS}E_{v}$ across all variables (e.g., 1.35 vs. 1.50 for DMCo)—the NIR.QST still demonstrated impressive robustness and reliability, especially given its portability and field applicability.

These results confirm the PLS model's adaptability across spectrometers. Similar findings were reported by Bantadjan et al. (2020), with $R_{v}$ = 0.74–0.77 for StC using PLS on 200 Thai cassava samples, and Chaiareekitwat et al. (2024), with $R_{v}$ = 0.98 and $\mathrm{RMS}E_{v}$ = 0.68 for DMCo. Altogether, our findings affirm the effectiveness of PLS regression for predicting starch and dry matter in cassava, offering practical insights on model, sample, and instrument choices for breeding and quality programs.

Despite promising results, several important limitations should be acknowledged. The predictive models were developed on a Brazilian cassava population, so their generalizability to other genetic backgrounds and breeding programs remains to be demonstrated. Although our results indicate that portable spectrometers can achieve performance close to benchtop systems in many cases, the lower spectral resolution and signal‐to‐noise of some handheld devices can reduce prediction accuracy, particularly under variable field conditions, so caution is warranted when extrapolating performance. Rigorous sample pre‐processing and standardized sampling protocols remain essential, as differences in sample preparation, environmental variability, and inter‐instrument calibration can all degrade model transferability and must be addressed through calibration‐transfer strategies or expanded calibration sets. Finally, independent validation on diverse cassava populations, multiple seasons, and a wider range of field conditions is required to confirm robustness and to quantify how device choice and operational constraints affect real‐world performance. Studies using handheld NIR for cassava phenotyping have shown feasibility but also highlight the need for broader testing before routine deployment in breeding programs.

### 4.5 Prospects of NIRS for Cassava Breeding

NIR spectroscopy has become a key tool in crop breeding, including cassava. Its non‐destructive nature, speed, and low operational cost offer a practical alternative to conventional laboratory methods for evaluating traits like starch and DMC, cyanogenic potential, and pasting properties (Abubakar et al. 2024; Chaiareekitwat et al. 2024; Maraphum et al. 2020). These advantages make NIRS ideal for early‐stage selection, where rapid evaluation of many genotypes is essential.

Currently, traits such as starch and dry matter are typically assessed only in final selection stages due to the cost and labor involved in standard methods. Oven‐drying, the gold standard, though accurate, demands time and infrastructure, creating bottlenecks in high‐throughput programs. Gravimetric methods, based on empirical equations, often yield inconsistent or inaccurate results. These limitations hinder scalability and may lead to the loss of superior genotypes due to unreliable data. The use of NIRS in early breeding stages represents a breakthrough, enabling faster, cost‐effective, and accurate trait assessments (Hershberger et al. 2022; Ikeogu et al. 2017; Lu et al. 2006). Additionally, NIRS allows longitudinal monitoring of traits throughout the development cycle, improving selection accuracy and resource use in breeding pipelines.

From a breeding perspective, the predictive power of NIRS contributes directly to greater genetic gains. Enhancing both accuracy and speed of evaluation allows breeders to more efficiently identify superior genotypes and accelerate genetic progress. This is crucial for crops with long cycles, helping reduce the time to develop improved varieties. Moreover, NIRS supports the evaluation of multiple traits simultaneously, promoting a broader and more effective selection process.

NIRS adoption also leads to significant cost savings. Traditional methods involve substantial labor, equipment, and consumables. Replacing them with NIRS reduces expenses related to sampling, lab analyses, and field testing. Its high‐throughput capacity enables evaluation of larger sample sizes, generating more robust data at lower cost—an especially important benefit for large‐scale programs where phenotyping costs are a major bottleneck.

Operational efficiency is also enhanced with NIRS. Its rapid analysis—either in‐field or in the lab—reduces delays in data collection and decision‐making. Real‐time monitoring of phenotypic changes further supports dynamic management, enabling timely adjustments in selection strategies. This agility leads to smoother operations, better resource allocation, and greater productivity. Practically, portable NIRS devices, although slightly less precise than bench‐top systems, offer notable advantages for breeding programs. They enable fast, direct field measurements, eliminating complex sample preparation and reducing logistical constraints. With moderate upfront costs, intuitive operation, and minimal maintenance, limited to periodic calibration and basic cleaning, these devices support high‐throughput evaluation of large populations across multiple sites, accelerating selection decisions and enhancing the efficiency and effectiveness of breeding programs.

Choosing between benchtop and portable devices is key. Benchtop spectrometers offer superior resolution, suitable for controlled settings. However, transporting samples can cause degradation, affecting data reliability. In contrast, portable spectrometers, though less precise, allow real‐time in‐field measurements with reduced risk of sample alteration, making them ideal for remote sites or stations lacking laboratory infrastructure.

Among the most impactful NIRS applications in cassava is starch estimation—vital for food, feed, and bioenergy. Since NIRS can estimate starch rapidly and non‐destructively, it enables early identification of genotypes with superior content. Its power is amplified when paired with machine learning models like PLS regression (Alamu et al. 2021) and XGB (Sousa et al. 2023), which enhance prediction accuracy and allow integration with genotypic data to model complex traits not directly measurable.

The integration of NIRS with complementary tools—such as molecular genotyping and advanced predictive modeling—marks a new phase in cassava improvement. This combined approach accelerates the development of high‐yielding, disease‐resistant cultivars with better starch quality. Ultimately, the fusion of spectroscopy and data‐driven breeding promises to revolutionize cassava research, making it faster, more precise, and sustainable.

## 5 Conclusions

This study demonstrated substantial variability in NIR model performance across prediction algorithms, devices, and sample types, emphasizing the importance of selecting the optimal combination of model, instrument, and sample preparation for accurate predictions.

Sample preparation was a key factor: mashed samples, due to their homogeneity, consistently reduced internal variability and yielded superior performance, while fresh roots showed lower accuracy due to structural heterogeneity. Among devices, the benchtop NIR.N500 provided the highest accuracy, particularly with PLS regression (e.g., $R_{C}$ = 0.98, $\mathrm{RMS}E_{C}$ = 0.81 for DMCo). The handheld NIR.QST also produced competitive results when paired with mashed samples ($R_{C}$​ up to 0.95 for DMCo), offering practical advantages such as affordability, portability, and rapid in‐field measurements that reduce sample degradation.

Independent and external validations confirmed these trends, demonstrating the robustness and reproducibility of the approach. Overall, our findings highlight that effective NIR phenotyping in cassava depends not solely on the spectrometer or algorithm, but on the strategic integration of device, model, and sample processing. This integrated approach enables scalable, cost‐effective, and efficient breeding pipelines, accelerating the identification of superior genotypes and the development of improved cultivars.

## Author Contributions

**Paulo Henrique Ramos Guimarães**: investigation, writing – original draft, methodology, validation, visualization, software, formal analysis, data curation. **Massaine Bandeira e Sousa**: investigation, methodology, validation, visualization, software, formal analysis, data curation, writing – review and editing. **Marcos de Souza Campos**: writing – review and editing, visualization, investigation. **Cinara Fernanda Garcia Morales**: investigation, visualization, writing – review and editing. **Eder Jorge de Oliveira**: funding acquisition, writing – review and editing, project administration, supervision, resources, conceptualization.

## Funding

Paulo Henrique Ramos Guimarães: Empresa Brasileira de Pesquisa Agropecuária. Grant number: 20.18.01.012.00.00. Massaine Bandeira e Sousa: Empresa Brasileira de Pesquisa Agropecuária. Grant number: 20.18.01.012.00.00. Eder Jorge de Oliveira: CNPq (Conselho Nacional de Desenvolvimento Científico e Tecnológico). Grant numbers: 310980/2021‐6 and 402422/2023‐6; FAPESB (Fundação de Amparo à Pesquisa do Estado da Bahia). Grant number: Pronem 15/2014.

This work was partially funded by the United Kingdom's Foreign, Commonwealth and Development Office (FCDO) and the Bill and Melinda Gates Foundation. Grant INV‐007637. Under the grant conditions of the Foundation, a Creative Commons Attribution 4.0 Generic License has already been assigned to the Author Accepted Manuscript version that might arise from this submission. The funder provided support in the form of a fellowship and funds for the research but did not have any additional role in the study design, data collection and analysis, decision to publish, or preparation of the manuscript.

## Conflicts of Interest

The authors declare no conflicts of interest.

## Data Availability

The raw data supporting the conclusions of this article will be made available by the authors, without undue reservation.
